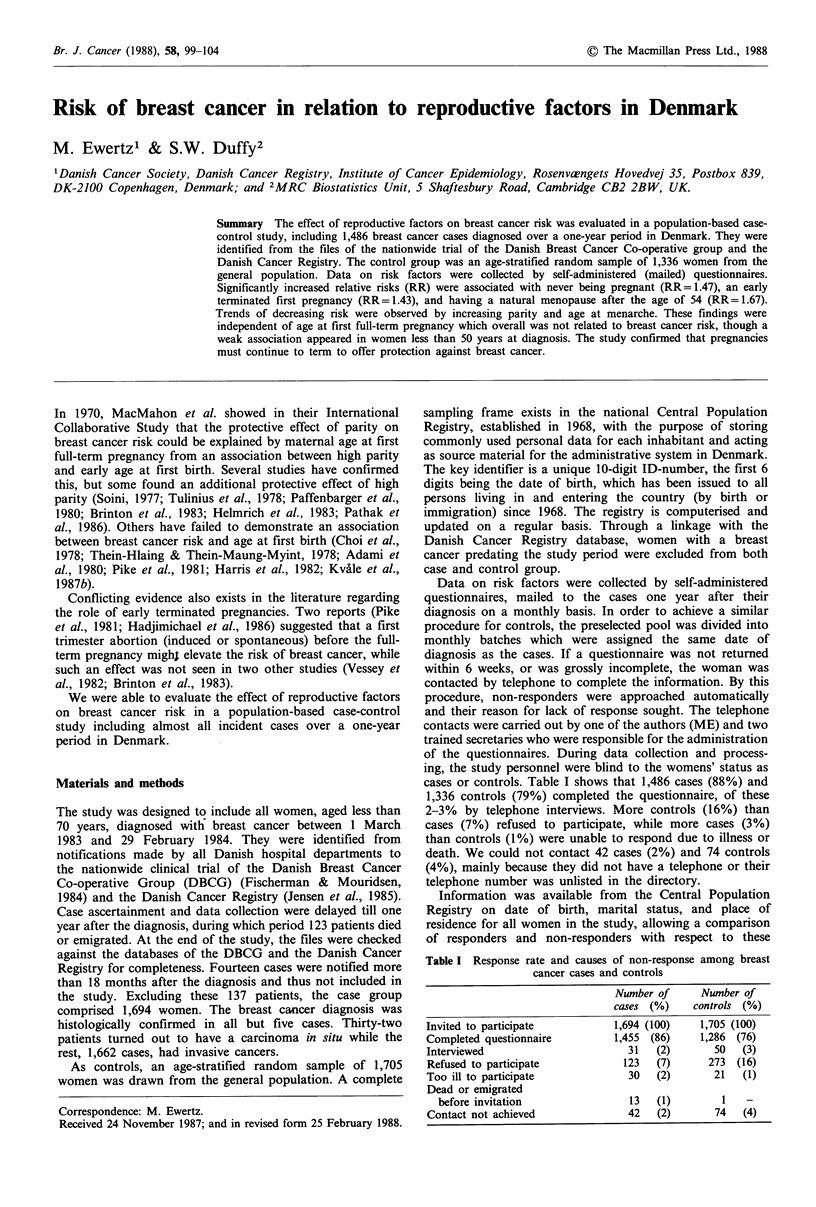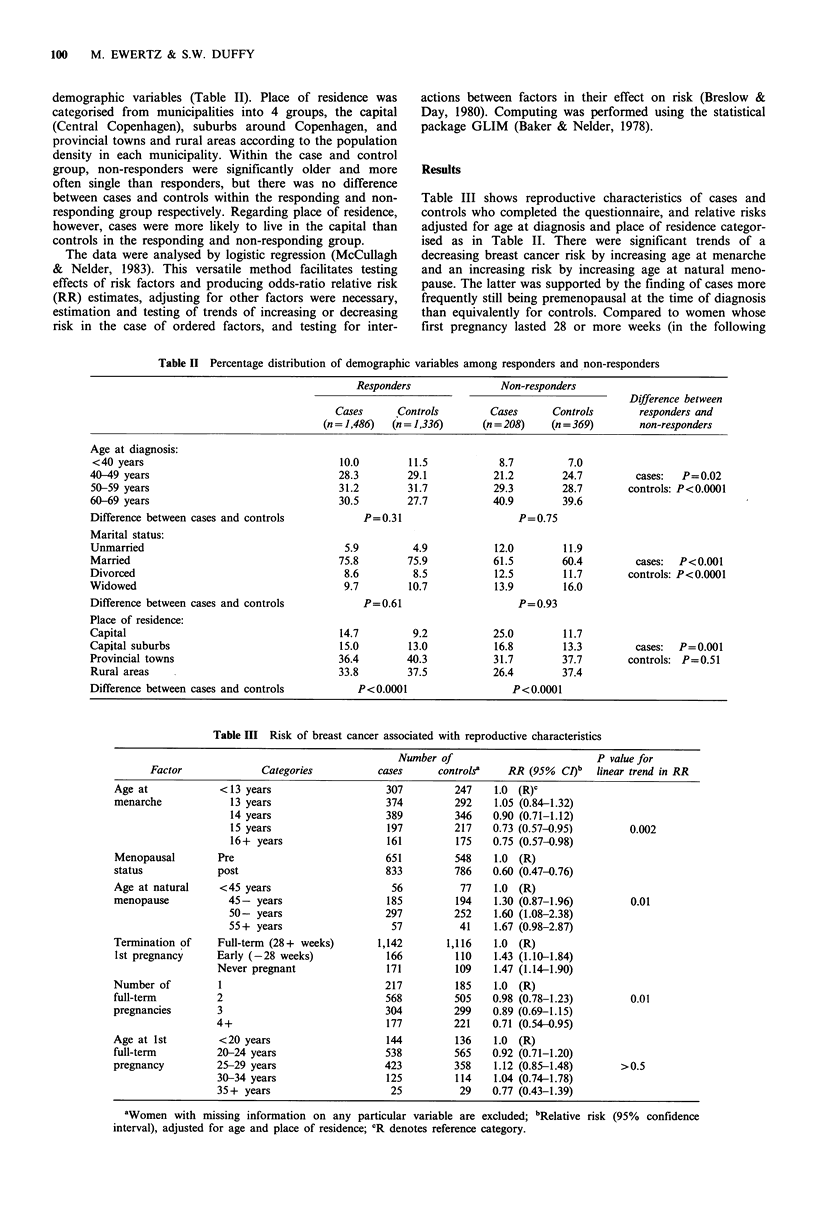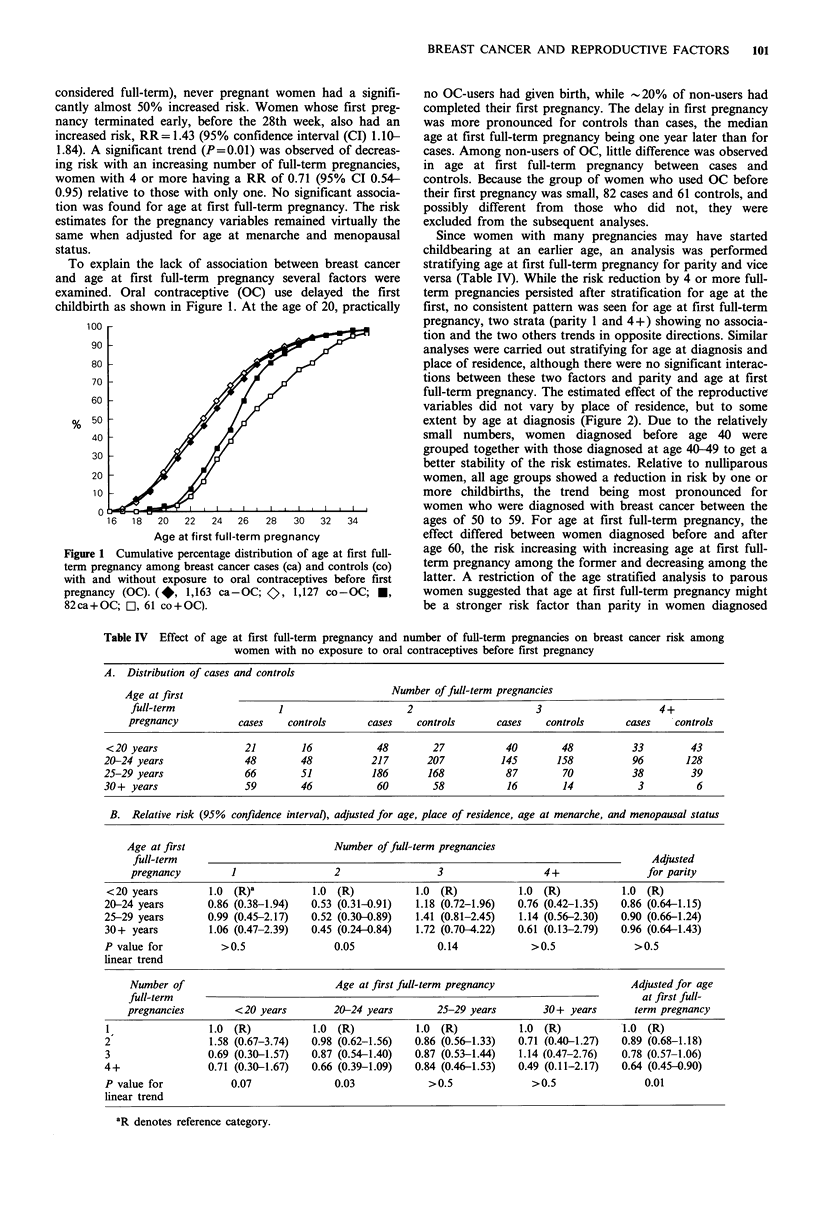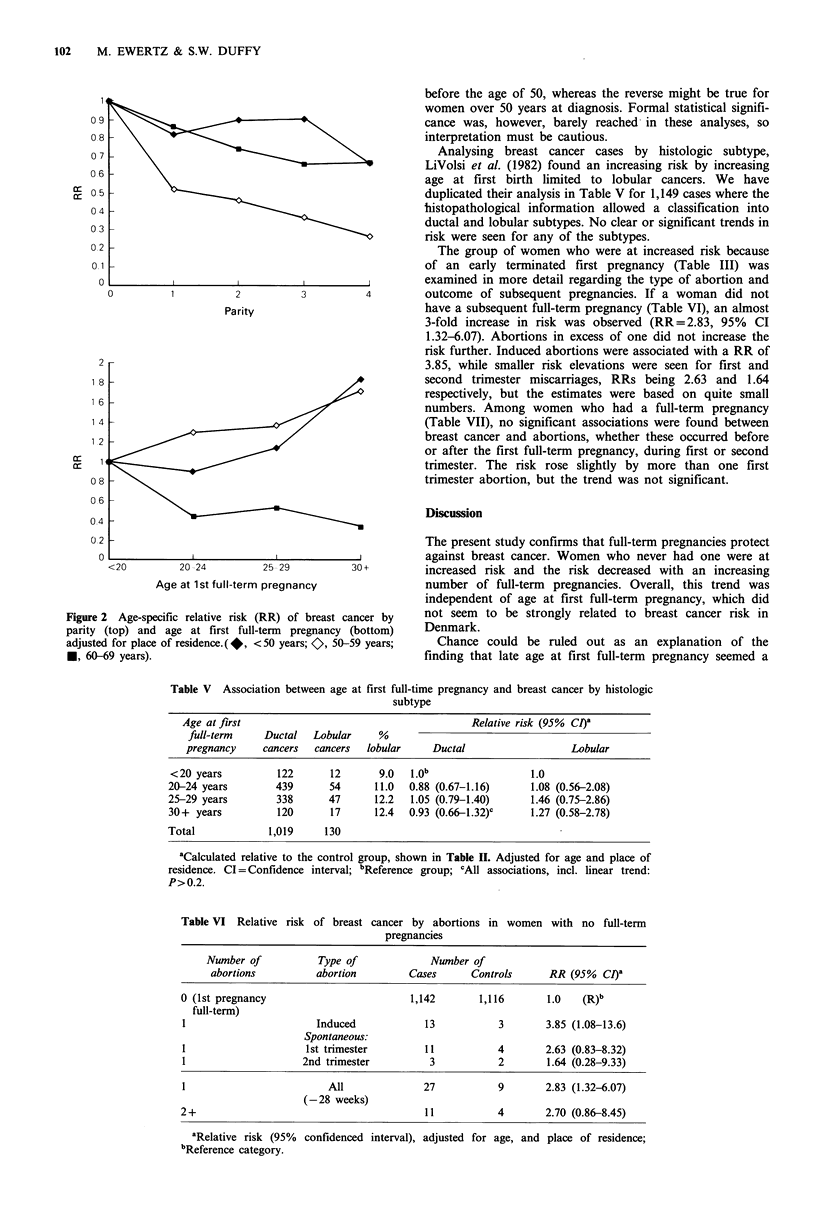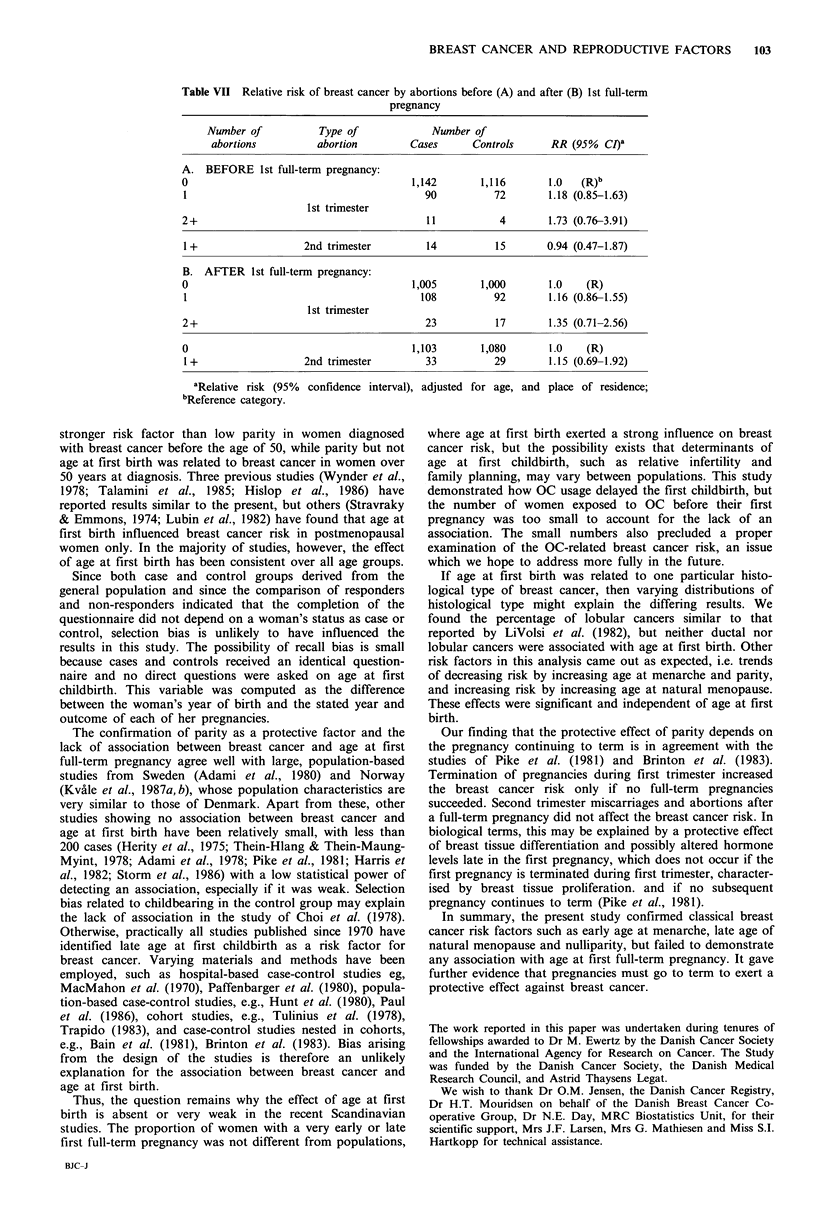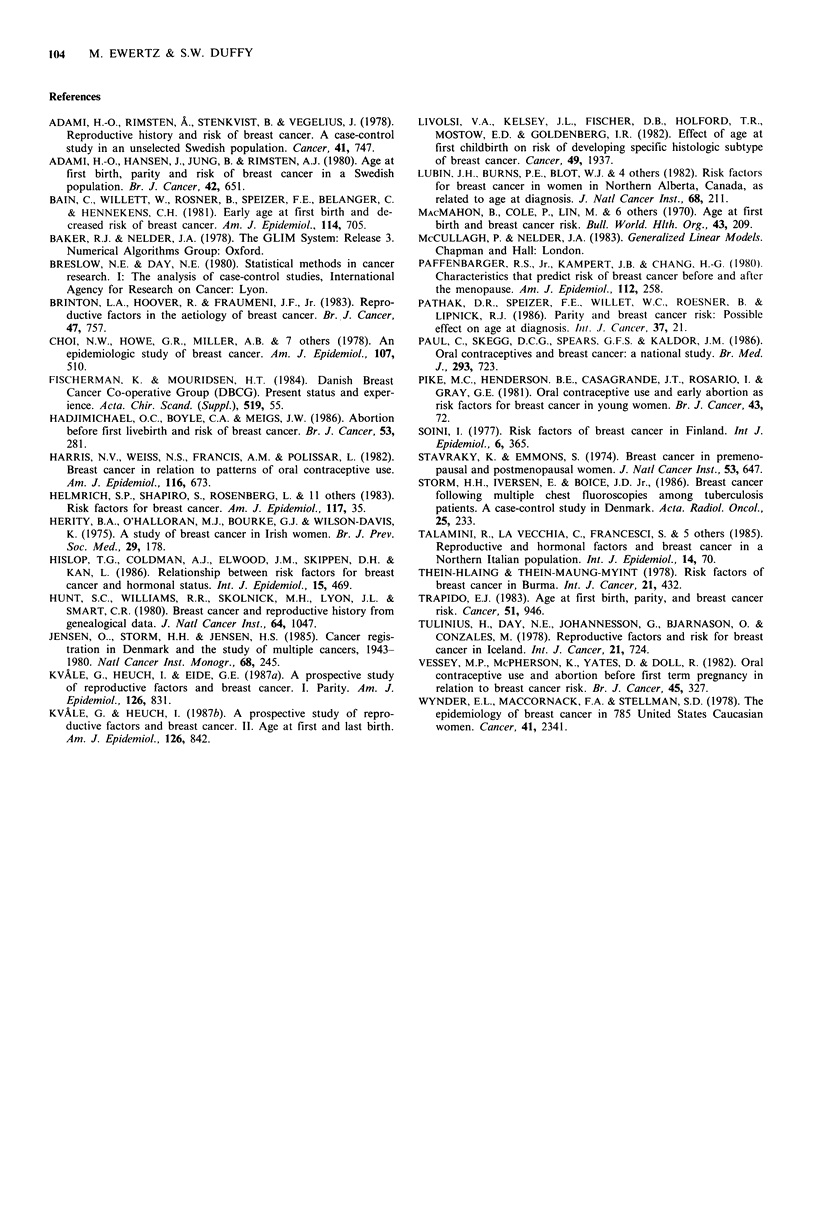# Risk of breast cancer in relation to reproductive factors in Denmark.

**DOI:** 10.1038/bjc.1988.172

**Published:** 1988-07

**Authors:** M. Ewertz, S. W. Duffy

**Affiliations:** Danish Cancer Society, Institute of Cancer Epidemiology, Copenhagen, Denmark.

## Abstract

The effect of reproductive factors on breast cancer risk was evaluated in a population-based case-control study, including 1,486 breast cancer cases diagnosed over a one-year period in Denmark. They were identified from the files of the nationwide trial of the Danish Breast Cancer Co-operative group and the Danish Cancer Registry. The control group was an age-stratified random sample of 1,336 women from the general population. Data on risk factors were collected by self-administered (mailed) questionnaires. Significantly increased relative risks (RR) were associated with never being pregnant (RR = 1.47), an early terminated first pregnancy (RR = 1.43), and having a natural menopause after the age of 54 (RR = 1.67). Trends of decreasing risk were observed by increasing parity and age at menarche. These findings were independent of age at first full-term pregnancy which overall was not related to breast cancer risk, though a weak association appeared in women less than 50 years at diagnosis. The study confirmed that pregnancies must continue to term to offer protection against breast cancer.


Br. J. Cancer (1988), 58, 99–104 © The Macmillan Press Ltd., 1988

### Risk of breast cancer in relation to reproductive factors in Denmark

M. Ewertz[1] & S.W. Duffy[2]

[1]*Danish Cancer Society, Danish Cancer Registry, Institute of Cancer Epidemiology, Rosenvængets Hovedvej 35, Postbox 839, DK-2100 Copenhagen, Denmark; and* [2]*MRC Biostatistics Unit, 5 Shaftesbury Road, Cambridge CB2 2BW, UK.*

**Summary** The effect of reproductive factors on breast cancer risk was evaluated in a population-based case-control study, including 1,486 breast cancer cases diagnosed over a one-year period in Denmark. They were identified from the files of the nationwide trial of the Danish Breast Cancer Co-operative group and the Danish Cancer Registry. The control group was an age-stratified random sample of 1,336 women from the general population. Data on risk factors were collected by self-administered (mailed) questionnaires. Significantly increased relative risks (RR) were associated with never being pregnant (RR = 1.47), an early terminated first pregnancy (RR = 1.43), and having a natural menopause after the age of 54 (RR = 1.67). Trends of decreasing risk were observed by increasing parity and age at menarche. These findings were independent of age at first full-term pregnancy which overall was not related to breast cancer risk, though a weak association appeared in women less than 50 years at diagnosis. The study confirmed that pregnancies must continue to term to offer protection against breast cancer.

In 1970, MacMahon *et al.* showed in their International Collaborative Study that the protective effect of parity on breast cancer risk could be explained by maternal age at first full-term pregnancy from an association between high parity and early age at first birth. Several studies have confirmed this, but some found an additional protective effect of high parity (Soini, 1977; Tulinius *et al.*, 1978; Paffenbarger *et al.*, 1980; Brinton *et al.*, 1983; Helmrich *et al.*, 1983; Pathak *et al.*, 1986). Others have failed to demonstrate an association between breast cancer risk and age at first birth (Choi *et al.*, 1978; Thein-Hlaing & Thein-Maung-Myint, 1978; Adami *et al.*, 1980; Pike *et al.*, 1981; Harris *et al.*, 1982; Kvåle *et al.*, 1987*b*).

Conflicting evidence also exists in the literature regarding the role of early terminated pregnancies. Two reports (Pike *et al.*, 1981; Hadjimichael *et al.*, 1986) suggested that a first trimester abortion (induced or spontaneous) before the full-term pregnancy might elevate the risk of breast cancer, while such an effect was not seen in two other studies (Vessey *et al.*, 1982; Brinton *et al.*, 1983).

We were able to evaluate the effect of reproductive factors on breast cancer risk in a population-based case-control study including almost all incident cases over a one-year period in Denmark.

### Materials and methods

The study was designed to include all women, aged less than 70 years, diagnosed with breast cancer between 1 March 1983 and 29 February 1984. They were identified from notifications made by all Danish hospital departments to the nationwide clinical trial of the Danish Breast Cancer Co-operative Group (DBCG) (Fischerman & Mouridsen, 1984) and the Danish Cancer Registry (Jensen *et al.*, 1985). Case ascertainment and data collection were delayed till one year after the diagnosis, during which period 123 patients died or emigrated. At the end of the study, the files were checked against the databases of the DBCG and the Danish Cancer Registry for completeness. Fourteen cases were notified more than 18 months after the diagnosis and thus not included in the study. Excluding these 137 patients, the case group comprised 1,694 women. The breast cancer diagnosis was histologically confirmed in all but five cases. Thirty-two patients turned out to have a carcinoma *in situ* while the rest, 1,662 cases, had invasive cancers.

As controls, an age-stratified random sample of 1,705 women was drawn from the general population. A complete sampling frame exists in the national Central Population Registry, established in 1968, with the purpose of storing commonly used personal data for each inhabitant and acting as source material for the administrative system in Denmark. The key identifier is a unique 10-digit ID-number, the first 6 digits being the date of birth, which has been issued to all persons living in and entering the country (by birth or immigration) since 1968. The registry is computerised and updated on a regular basis. Through a linkage with the Danish Cancer Registry database, women with a breast cancer predating the study period were excluded from both case and control group.

Data on risk factors were collected by self-administered questionnaires, mailed to the cases one year after their diagnosis on a monthly basis. In order to achieve a similar procedure for controls, the preselected pool was divided into monthly batches which were assigned the same date of diagnosis as the cases. If a questionnaire was not returned within 6 weeks, or was grossly incomplete, the woman was contacted by telephone to complete the information. By this procedure, non-responders were approached automatically and their reason for lack of response sought. The telephone contacts were carried out by one of the authors (ME) and two trained secretaries who were responsible for the administration of the questionnaires. During data collection and processing, the study personnel were blind to the womens' status as cases or controls. Table I shows that 1,486 cases (88%) and 1,336 controls (79%) completed the questionnaire, of these 2–3% by telephone interviews. More controls (16%) than cases (7%) refused to participate, while more cases (3%) than controls (1%) were unable to respond due to illness or death. We could not contact 42 cases (2%) and 74 controls (4%), mainly because they did not have a telephone or their telephone number was unlisted in the directory.

Information was available from the Central Population Registry on date of birth, marital status, and place of residence for all women in the study, allowing a comparison of responders and non-responders with respect to these

**Table I** Response rate and causes of non-response among breast cancer cases and controls

| | *Number of cases* | (%) | *Number of controls* | (%) |
|---|---|---|---|---|
| Invited to participate | 1,694 | (100) | 1,705 | (100) |
| Completed questionnaire | 1,455 | (86) | 1,286 | (76) |
| Interviewed | 31 | (2) | 50 | (3) |
| Refused to participate | 123 | (7) | 273 | (16) |
| Too ill to participate | 30 | (2) | 21 | (1) |
| Dead or emigrated before invitation | 13 | (1) | 1 | – |
| Contact not achieved | 42 | (2) | 74 | (4) |

Correspondence: M. Ewertz.
Received 24 November 1987; and in revised form 25 February 1988.

demographic variables (Table II). Place of residence was categorised from municipalities into 4 groups, the capital (Central Copenhagen), suburbs around Copenhagen, and provincial towns and rural areas according to the population density in each municipality. Within the case and control group, non-responders were significantly older and more often single than responders, but there was no difference between cases and controls within the responding and non-responding group respectively. Regarding place of residence, however, cases were more likely to live in the capital than controls in the responding and non-responding group.

The data were analysed by logistic regression (McCullagh & Nelder, 1983). This versatile method facilitates testing effects of risk factors and producing odds-ratio relative risk (RR) estimates, adjusting for other factors were necessary, estimation and testing of trends of increasing or decreasing risk in the case of ordered factors, and testing for interactions between factors in their effect on risk (Breslow & Day, 1980). Computing was performed using the statistical package GLIM (Baker & Nelder, 1978).

### Results

Table III shows reproductive characteristics of cases and controls who completed the questionnaire, and relative risks adjusted for age at diagnosis and place of residence categorised as in Table II. There were significant trends of a decreasing breast cancer risk by increasing age at menarche and an increasing risk by increasing age at natural menopause. The latter was supported by the finding of cases more frequently still being premenopausal at the time of diagnosis than equivalently for controls. Compared to women whose first pregnancy lasted 28 or more weeks (in the following

**Table II** Percentage distribution of demographic variables among responders and non-responders

| | *Responders* | | *Non-responders* | | *Difference between responders and non-responders* |
|---|---|---|---|---|---|
| | *Cases (n=1,486)* | *Controls (n=1,336)* | *Cases (n=208)* | *Controls (n=369)* | |
| Age at diagnosis: | | | | | |
| <40 years | 10.0 | 11.5 | 8.7 | 7.0 | |
| 40–49 years | 28.3 | 29.1 | 21.2 | 24.7 | cases: $P=0.02$ |
| 50–59 years | 31.2 | 31.7 | 29.3 | 28.7 | controls: $P<0.0001$ |
| 60–69 years | 30.5 | 27.7 | 40.9 | 39.6 | |
| Difference between cases and controls | $P=0.31$ | | $P=0.75$ | | |
| Marital status: | | | | | |
| Unmarried | 5.9 | 4.9 | 12.0 | 11.9 | |
| Married | 75.8 | 75.9 | 61.5 | 60.4 | cases: $P<0.001$ |
| Divorced | 8.6 | 8.5 | 12.5 | 11.7 | controls: $P<0.0001$ |
| Widowed | 9.7 | 10.7 | 13.9 | 16.0 | |
| Difference between cases and controls | $P=0.61$ | | $P=0.93$ | | |
| Place of residence: | | | | | |
| Capital | 14.7 | 9.2 | 25.0 | 11.7 | |
| Capital suburbs | 15.0 | 13.0 | 16.8 | 13.3 | cases: $P=0.001$ |
| Provincial towns | 36.4 | 40.3 | 31.7 | 37.7 | controls: $P=0.51$ |
| Rural areas | 33.8 | 37.5 | 26.4 | 37.4 | |
| Difference between cases and controls | $P<0.0001$ | | $P<0.0001$ | | |

**Table III** Risk of breast cancer associated with reproductive characteristics

| *Factor* | *Categories* | *Number of cases* | *Number of controls*[a] | *RR (95% CI)*[b] | *P value for linear trend in RR* |
|---|---|---|---|---|---|
| Age at menarche | <13 years | 307 | 247 | 1.0 (R)[c] | |
| | 13 years | 374 | 292 | 1.05 (0.84–1.32) | |
| | 14 years | 389 | 346 | 0.90 (0.71–1.12) | |
| | 15 years | 197 | 217 | 0.73 (0.57–0.95) | 0.002 |
| | 16+ years | 161 | 175 | 0.75 (0.57–0.98) | |
| Menopausal status | Pre | 651 | 548 | 1.0 (R) | |
| | post | 833 | 786 | 0.60 (0.47–0.76) | |
| Age at natural menopause | <45 years | 56 | 77 | 1.0 (R) | |
| | 45– years | 185 | 194 | 1.30 (0.87–1.96) | 0.01 |
| | 50– years | 297 | 252 | 1.60 (1.08–2.38) | |
| | 55+ years | 57 | 41 | 1.67 (0.98–2.87) | |
| Termination of 1st pregnancy | Full-term (28+ weeks) | 1,142 | 1,116 | 1.0 (R) | |
| | Early (–28 weeks) | 166 | 110 | 1.43 (1.10–1.84) | |
| | Never pregnant | 171 | 109 | 1.47 (1.14–1.90) | |
| Number of full-term pregnancies | 1 | 217 | 185 | 1.0 (R) | |
| | 2 | 568 | 505 | 0.98 (0.78–1.23) | 0.01 |
| | 3 | 304 | 299 | 0.89 (0.69–1.15) | |
| | 4+ | 177 | 221 | 0.71 (0.54–0.95) | |
| Age at 1st full-term pregnancy | <20 years | 144 | 136 | 1.0 (R) | |
| | 20–24 years | 538 | 565 | 0.92 (0.71–1.20) | |
| | 25–29 years | 423 | 358 | 1.12 (0.85–1.48) | >0.5 |
| | 30–34 years | 125 | 114 | 1.04 (0.74–1.78) | |
| | 35+ years | 25 | 29 | 0.77 (0.43–1.39) | |

[a]Women with missing information on any particular variable are excluded; [b]Relative risk (95% confidence interval), adjusted for age and place of residence; [c]R denotes reference category.

considered full-term), never pregnant women had a significantly almost 50% increased risk. Women whose first pregnancy terminated early, before the 28th week, also had an increased risk, RR = 1.43 (95% confidence interval (CI) 1.10–1.84). A significant trend ($P = 0.01$) was observed of decreasing risk with an increasing number of full-term pregnancies, women with 4 or more having a RR of 0.71 (95% CI 0.54–0.95) relative to those with only one. No significant association was found for age at first full-term pregnancy. The risk estimates for the pregnancy variables remained virtually the same when adjusted for age at menarche and menopausal status.

To explain the lack of association between breast cancer and age at first full-term pregnancy several factors were examined. Oral contraceptive (OC) use delayed the first childbirth as shown in Figure 1. At the age of 20, practically

**Figure 1** Cumulative percentage distribution of age at first full-term pregnancy among breast cancer cases (ca) and controls (co) with and without exposure to oral contraceptives before first pregnancy (OC). (◆, 1,163 ca − OC; ◇, 1,127 co − OC; ■, 82 ca + OC; □, 61 co + OC).

no OC-users had given birth, while ~20% of non-users had completed their first pregnancy. The delay in first pregnancy was more pronounced for controls than cases, the median age at first full-term pregnancy being one year later than for cases. Among non-users of OC, little difference was observed in age at first full-term pregnancy between cases and controls. Because the group of women who used OC before their first pregnancy was small, 82 cases and 61 controls, and possibly different from those who did not, they were excluded from the subsequent analyses.

Since women with many pregnancies may have started childbearing at an earlier age, an analysis was performed stratifying age at first full-term pregnancy for parity and vice versa (Table IV). While the risk reduction by 4 or more full-term pregnancies persisted after stratification for age at the first, no consistent pattern was seen for age at first full-term pregnancy, two strata (parity 1 and 4+) showing no association and the two others trends in opposite directions. Similar analyses were carried out stratifying for age at diagnosis and place of residence, although there were no significant interactions between these two factors and parity and age at first full-term pregnancy. The estimated effect of the reproductive variables did not vary by place of residence, but to some extent by age at diagnosis (Figure 2). Due to the relatively small numbers, women diagnosed before age 40 were grouped together with those diagnosed at age 40–49 to get a better stability of the risk estimates. Relative to nulliparous women, all age groups showed a reduction in risk by one or more childbirths, the trend being most pronounced for women who were diagnosed with breast cancer between the ages of 50 to 59. For age at first full-term pregnancy, the effect differed between women diagnosed before and after age 60, the risk increasing with increasing age at first full-term pregnancy among the former and decreasing among the latter. A restriction of the age stratified analysis to parous women suggested that age at first full-term pregnancy might be a stronger risk factor than parity in women diagnosed

**Table IV** Effect of age at first full-term pregnancy and number of full-term pregnancies on breast cancer risk among women with no exposure to oral contraceptives before first pregnancy

*A. Distribution of cases and controls*

| *Age at first full-term pregnancy* | *Number of full-term pregnancies* | | | | | | | |
|---|---|---|---|---|---|---|---|---|
| | *1* | | *2* | | *3* | | *4+* | |
| | *cases* | *controls* | *cases* | *controls* | *cases* | *controls* | *cases* | *controls* |
| *<20 years* | *21* | *16* | *48* | *27* | *40* | *48* | *33* | *43* |
| *20–24 years* | *48* | *48* | *217* | *207* | *145* | *158* | *96* | *128* |
| *25–29 years* | *66* | *51* | *186* | *168* | *87* | *70* | *38* | *39* |
| *30+ years* | *59* | *46* | *60* | *58* | *16* | *14* | *3* | *6* |

*B. Relative risk (95% confidence interval), adjusted for age, place of residence, age at menarche, and menopausal status*

| *Age at first full-term pregnancy* | *Number of full-term pregnancies* | | | | *Adjusted for parity* |
|---|---|---|---|---|---|
| | *1* | *2* | *3* | *4+* | |
| <20 years | 1.0 (R)[a] | 1.0 (R) | 1.0 (R) | 1.0 (R) | 1.0 (R) |
| 20–24 years | 0.86 (0.38–1.94) | 0.53 (0.31–0.91) | 1.18 (0.72–1.96) | 0.76 (0.42–1.35) | 0.86 (0.64–1.15) |
| 25–29 years | 0.99 (0.45–2.17) | 0.52 (0.30–0.89) | 1.41 (0.81–2.45) | 1.14 (0.56–2.30) | 0.90 (0.66–1.24) |
| 30+ years | 1.06 (0.47–2.39) | 0.45 (0.24–0.84) | 1.72 (0.70–4.22) | 0.61 (0.13–2.79) | 0.96 (0.64–1.43) |
| *P* value for linear trend | >0.5 | 0.05 | 0.14 | >0.5 | >0.5 |

| *Number of full-term pregnancies* | *Age at first full-term pregnancy* | | | | *Adjusted for age at first full-term pregnancy* |
|---|---|---|---|---|---|
| | *<20 years* | *20–24 years* | *25–29 years* | *30+ years* | |
| 1 | 1.0 (R) | 1.0 (R) | 1.0 (R) | 1.0 (R) | 1.0 (R) |
| 2 | 1.58 (0.67–3.74) | 0.98 (0.62–1.56) | 0.86 (0.56–1.33) | 0.71 (0.40–1.27) | 0.89 (0.68–1.18) |
| 3 | 0.69 (0.30–1.57) | 0.87 (0.54–1.40) | 0.87 (0.53–1.44) | 1.14 (0.47–2.76) | 0.78 (0.57–1.06) |
| 4+ | 0.71 (0.30–1.67) | 0.66 (0.39–1.09) | 0.84 (0.46–1.53) | 0.49 (0.11–2.17) | 0.64 (0.45–0.90) |
| *P* value for linear trend | 0.07 | 0.03 | >0.5 | >0.5 | 0.01 |

[a]R denotes reference category.

**Figure 2** Age-specific relative risk (RR) of breast cancer by parity (top) and age at first full-term pregnancy (bottom) adjusted for place of residence. (◆, <50 years; ◇, 50–59 years; ■, 60–69 years).

before the age of 50, whereas the reverse might be true for women over 50 years at diagnosis. Formal statistical significance was, however, barely reached in these analyses, so interpretation must be cautious.

Analysing breast cancer cases by histologic subtype, LiVolsi *et al.* (1982) found an increasing risk by increasing age at first birth limited to lobular cancers. We have duplicated their analysis in Table V for 1,149 cases where the histopathological information allowed a classification into ductal and lobular subtypes. No clear or significant trends in risk were seen for any of the subtypes.

The group of women who were at increased risk because of an early terminated first pregnancy (Table III) was examined in more detail regarding the type of abortion and outcome of subsequent pregnancies. If a woman did not have a subsequent full-term pregnancy (Table VI), an almost 3-fold increase in risk was observed (RR = 2.83, 95% CI 1.32–6.07). Abortions in excess of one did not increase the risk further. Induced abortions were associated with a RR of 3.85, while smaller risk elevations were seen for first and second trimester miscarriages, RRs being 2.63 and 1.64 respectively, but the estimates were based on quite small numbers. Among women who had a full-term pregnancy (Table VII), no significant associations were found between breast cancer and abortions, whether these occurred before or after the first full-term pregnancy, during first or second trimester. The risk rose slightly by more than one first trimester abortion, but the trend was not significant.

### Discussion

The present study confirms that full-term pregnancies protect against breast cancer. Women who never had one were at increased risk and the risk decreased with an increasing number of full-term pregnancies. Overall, this trend was independent of age at first full-term pregnancy, which did not seem to be strongly related to breast cancer risk in Denmark.

Chance could be ruled out as an explanation of the finding that late age at first full-term pregnancy seemed a

**Table V** Association between age at first full-time pregnancy and breast cancer by histologic subtype

| *Age at first full-term pregnancy* | *Ductal cancers* | *Lobular cancers* | *% lobular* | *Relative risk (95% CI)*[a] *Ductal* | *Lobular* |
|---|---|---|---|---|---|
| <20 years | 122 | 12 | 9.0 | 1.0[b] | 1.0 |
| 20–24 years | 439 | 54 | 11.0 | 0.88 (0.67–1.16) | 1.08 (0.56–2.08) |
| 25–29 years | 338 | 47 | 12.2 | 1.05 (0.79–1.40) | 1.46 (0.75–2.86) |
| 30+ years | 120 | 17 | 12.4 | 0.93 (0.66–1.32)[c] | 1.27 (0.58–2.78) |
| Total | 1,019 | 130 | | | |

[a]Calculated relative to the control group, shown in **Table II**. Adjusted for age and place of residence. CI = Confidence interval; [b]Reference group; [c]All associations, incl. linear trend: $P > 0.2$.

**Table VI** Relative risk of breast cancer by abortions in women with no full-term pregnancies

| *Number of abortions* | *Type of abortion* | *Number of Cases* | *Controls* | *RR (95% CI)*[a] |
|---|---|---|---|---|
| 0 (1st pregnancy full-term) | | 1,142 | 1,116 | 1.0 (R)[b] |
| 1 | Induced | 13 | 3 | 3.85 (1.08–13.6) |
| | *Spontaneous:* | | | |
| 1 | 1st trimester | 11 | 4 | 2.63 (0.83–8.32) |
| 1 | 2nd trimester | 3 | 2 | 1.64 (0.28–9.33) |
| 1 | All (−28 weeks) | 27 | 9 | 2.83 (1.32–6.07) |
| 2+ | | 11 | 4 | 2.70 (0.86–8.45) |

[a]Relative risk (95% confidenced interval), adjusted for age, and place of residence; [b]Reference category.

**Table VII** Relative risk of breast cancer by abortions before (A) and after (B) 1st full-term pregnancy

| *Number of abortions* | *Type of abortion* | *Number of Cases* | *Number of Controls* | *RR (95% CI)*[a] |
|---|---|---|---|---|
| A. BEFORE 1st full-term pregnancy: | | | | |
| 0 | | 1,142 | 1,116 | 1.0 (R)[b] |
| 1 | 1st trimester | 90 | 72 | 1.18 (0.85–1.63) |
| 2+ | | 11 | 4 | 1.73 (0.76–3.91) |
| 1+ | 2nd trimester | 14 | 15 | 0.94 (0.47–1.87) |
| B. AFTER 1st full-term pregnancy: | | | | |
| 0 | | 1,005 | 1,000 | 1.0 (R) |
| 1 | 1st trimester | 108 | 92 | 1.16 (0.86–1.55) |
| 2+ | | 23 | 17 | 1.35 (0.71–2.56) |
| 0 | | 1,103 | 1,080 | 1.0 (R) |
| 1+ | 2nd trimester | 33 | 29 | 1.15 (0.69–1.92) |

[a]Relative risk (95% confidence interval), adjusted for age, and place of residence; [b]Reference category.

stronger risk factor than low parity in women diagnosed with breast cancer before the age of 50, while parity but not age at first birth was related to breast cancer in women over 50 years at diagnosis. Three previous studies (Wynder *et al.*, 1978; Talamini *et al.*, 1985; Hislop *et al.*, 1986) have reported results similar to the present, but others (Stravraky & Emmons, 1974; Lubin *et al.*, 1982) have found that age at first birth influenced breast cancer risk in postmenopausal women only. In the majority of studies, however, the effect of age at first birth has been consistent over all age groups.

Since both case and control groups derived from the general population and since the comparison of responders and non-responders indicated that the completion of the questionnaire did not depend on a woman's status as case or control, selection bias is unlikely to have influenced the results in this study. The possibility of recall bias is small because cases and controls received an identical questionnaire and no direct questions were asked on age at first childbirth. This variable was computed as the difference between the woman's year of birth and the stated year and outcome of each of her pregnancies.

The confirmation of parity as a protective factor and the lack of association between breast cancer and age at first full-term pregnancy agree well with large, population-based studies from Sweden (Adami *et al.*, 1980) and Norway (Kvåle *et al.*, 1987*a*, *b*), whose population characteristics are very similar to those of Denmark. Apart from these, other studies showing no association between breast cancer and age at first birth have been relatively small, with less than 200 cases (Herity *et al.*, 1975; Thein-Hlang & Thein-Maung-Myint, 1978; Adami *et al.*, 1978; Pike *et al.*, 1981; Harris *et al.*, 1982; Storm *et al.*, 1986) with a low statistical power of detecting an association, especially if it was weak. Selection bias related to childbearing in the control group may explain the lack of association in the study of Choi *et al.* (1978). Otherwise, practically all studies published since 1970 have identified late age at first childbirth as a risk factor for breast cancer. Varying materials and methods have been employed, such as hospital-based case-control studies eg, MacMahon *et al.* (1970), Paffenbarger *et al.* (1980), population-based case-control studies, e.g., Hunt *et al.* (1980), Paul *et al.* (1986), cohort studies, e.g., Tulinius *et al.* (1978), Trapido (1983), and case-control studies nested in cohorts, e.g., Bain *et al.* (1981), Brinton *et al.* (1983). Bias arising from the design of the studies is therefore an unlikely explanation for the association between breast cancer and age at first birth.

Thus, the question remains why the effect of age at first birth is absent or very weak in the recent Scandinavian studies. The proportion of women with a very early or late first full-term pregnancy was not different from populations, where age at first birth exerted a strong influence on breast cancer risk, but the possibility exists that determinants of age at first childbirth, such as relative infertility and family planning, may vary between populations. This study demonstrated how OC usage delayed the first childbirth, but the number of women exposed to OC before their first pregnancy was too small to account for the lack of an association. The small numbers also precluded a proper examination of the OC-related breast cancer risk, an issue which we hope to address more fully in the future.

If age at first birth was related to one particular histological type of breast cancer, then varying distributions of histological type might explain the differing results. We found the percentage of lobular cancers similar to that reported by LiVolsi *et al.* (1982), but neither ductal nor lobular cancers were associated with age at first birth. Other risk factors in this analysis came out as expected, i.e. trends of decreasing risk by increasing age at menarche and parity, and increasing risk by increasing age at natural menopause. These effects were significant and independent of age at first birth.

Our finding that the protective effect of parity depends on the pregnancy continuing to term is in agreement with the studies of Pike *et al.* (1981) and Brinton *et al.* (1983). Termination of pregnancies during first trimester increased the breast cancer risk only if no full-term pregnancies succeeded. Second trimester miscarriages and abortions after a full-term pregnancy did not affect the breast cancer risk. In biological terms, this may be explained by a protective effect of breast tissue differentiation and possibly altered hormone levels late in the first pregnancy, which does not occur if the first pregnancy is terminated during first trimester, characterised by breast tissue proliferation. and if no subsequent pregnancy continues to term (Pike *et al.*, 1981).

In summary, the present study confirmed classical breast cancer risk factors such as early age at menarche, late age of natural menopause and nulliparity, but failed to demonstrate any association with age at first full-term pregnancy. It gave further evidence that pregnancies must go to term to exert a protective effect against breast cancer.

The work reported in this paper was undertaken during tenures of fellowships awarded to Dr M. Ewertz by the Danish Cancer Society and the International Agency for Research on Cancer. The Study was funded by the Danish Cancer Society, the Danish Medical Research Council, and Astrid Thaysens Legat.

We wish to thank Dr O.M. Jensen, the Danish Cancer Registry, Dr H.T. Mouridsen on behalf of the Danish Breast Cancer Cooperative Group, Dr N.E. Day, MRC Biostatistics Unit, for their scientific support, Mrs J.F. Larsen, Mrs G. Mathiesen and Miss S.I. Hartkopp for technical assistance.

### References

ADAMI, H.-O., RIMSTEN, Å., STENKVIST, B. & VEGELIUS, J. (1978). Reproductive history and risk of breast cancer. A case-control study in an unselected Swedish population. *Cancer*, **41**, 747.

ADAMI, H.-O., HANSEN, J., JUNG, B. & RIMSTEN, A.J. (1980). Age at first birth, parity and risk of breast cancer in a Swedish population. *Br. J. Cancer*, **42**, 651.

BAIN, C., WILLETT, W., ROSNER, B., SPEIZER, F.E., BELANGER, C. & HENNEKENS, C.H. (1981). Early age at first birth and decreased risk of breast cancer. *Am. J. Epidemiol.*, **114**, 705.

BAKER, R.J. & NELDER, J.A. (1978). The GLIM System: Release 3. Numerical Algorithms Group: Oxford.

BRESLOW, N.E. & DAY, N.E. (1980). Statistical methods in cancer research. I: The analysis of case-control studies, International Agency for Research on Cancer: Lyon.

BRINTON, L.A., HOOVER, R. & FRAUMENI, J.F., Jr. (1983). Reproductive factors in the aetiology of breast cancer. *Br. J. Cancer*, **47**, 757.

CHOI, N.W., HOWE, G.R., MILLER, A.B. & 7 others (1978). An epidemiologic study of breast cancer. *Am. J. Epidemiol.*, **107**, 510.

FISCHERMAN, K. & MOURIDSEN, H.T. (1984). Danish Breast Cancer Co-operative Group (DBCG). Present status and experience. *Acta. Chir. Scand.* (*Suppl.*), **519**, 55.

HADJIMICHAEL, O.C., BOYLE, C.A. & MEIGS, J.W. (1986). Abortion before first livebirth and risk of breast cancer. *Br. J. Cancer*, **53**, 281.

HARRIS, N.V., WEISS, N.S., FRANCIS, A.M. & POLISSAR, L. (1982). Breast cancer in relation to patterns of oral contraceptive use. *Am. J. Epidemiol.*, **116**, 673.

HELMRICH, S.P., SHAPIRO, S., ROSENBERG, L. & 11 others (1983). Risk factors for breast cancer. *Am. J. Epidemiol.*, **117**, 35.

HERITY, B.A., O'HALLORAN, M.J., BOURKE, G.J. & WILSON-DAVIS, K. (1975). A study of breast cancer in Irish women. *Br. J. Prev. Soc. Med.*, **29**, 178.

HISLOP, T.G., COLDMAN, A.J., ELWOOD, J.M., SKIPPEN, D.H. & KAN, L. (1986). Relationship between risk factors for breast cancer and hormonal status. *Int. J. Epidemiol.*, **15**, 469.

HUNT, S.C., WILLIAMS, R.R., SKOLNICK, M.H., LYON, J.L. & SMART, C.R. (1980). Breast cancer and reproductive history from genealogical data. *J. Natl Cancer Inst.*, **64**, 1047.

JENSEN, O., STORM, H.H. & JENSEN, H.S. (1985). Cancer registration in Denmark and the study of multiple cancers, 1943–1980. *Natl Cancer Inst. Monogr.*, **68**, 245.

KVÅLE, G., HEUCH, I. & EIDE, G.E. (1987*a*). A prospective study of reproductive factors and breast cancer. I. Parity. *Am. J. Epidemiol.*, **126**, 831.

KVÅLE, G. & HEUCH, I. (1987*b*). A prospective study of reproductive factors and breast cancer. II. Age at first and last birth. *Am. J. Epidemiol.*, **126**, 842.

LIVOLSI, V.A., KELSEY, J.L., FISCHER, D.B., HOLFORD, T.R., MOSTOW, E.D. & GOLDENBERG, I.R. (1982). Effect of age at first childbirth on risk of developing specific histologic subtype of breast cancer. *Cancer*, **49**, 1937.

LUBIN, J.H., BURNS, P.E., BLOT, W.J. & 4 others (1982). Risk factors for breast cancer in women in Northern Alberta, Canada, as related to age at diagnosis. *J. Natl Cancer Inst.*, **68**, 211.

MACMAHON, B., COLE, P., LIN, M. & 6 others (1970). Age at first birth and breast cancer risk. *Bull. World. Hlth. Org.*, **43**, 209.

MCCULLAGH, P. & NELDER, J.A. (1983). *Generalized Linear Models*. Chapman and Hall: London.

PAFFENBARGER, R.S., Jr., KAMPERT, J.B. & CHANG, H.-G. (1980). Characteristics that predict risk of breast cancer before and after the menopause. *Am. J. Epidemiol.*, **112**, 258.

PATHAK, D.R., SPEIZER, F.E., WILLET, W.C., ROESNER, B. & LIPNICK, R.J. (1986). Parity and breast cancer risk: Possible effect on age at diagnosis. *Int. J. Cancer*, **37**, 21.

PAUL, C., SKEGG, D.C.G., SPEARS, G.F.S. & KALDOR, J.M. (1986). Oral contraceptives and breast cancer: a national study. *Br. Med. J.*, **293**, 723.

PIKE, M.C., HENDERSON, B.E., CASAGRANDE, J.T., ROSARIO, I. & GRAY, G.E. (1981). Oral contraceptive use and early abortion as risk factors for breast cancer in young women. *Br. J. Cancer*, **43**, 72.

SOINI, I. (1977). Risk factors of breast cancer in Finland. *Int J. Epidemiol.*, **6**, 365.

STAVRAKY, K. & EMMONS, S. (1974). Breast cancer in premenopausal and postmenopausal women. *J. Natl Cancer Inst.*, **53**, 647.

STORM, H.H., IVERSEN, E. & BOICE, J.D. Jr., (1986). Breast cancer following multiple chest fluoroscopies among tuberculosis patients. A case-control study in Denmark. *Acta. Radiol. Oncol.*, **25**, 233.

TALAMINI, R., LA VECCHIA, C., FRANCESCI, S. & 5 others (1985). Reproductive and hormonal factors and breast cancer in a Northern Italian population. *Int. J. Epidemiol.*, **14**, 70.

THEIN-HLAING & THEIN-MAUNG-MYINT (1978). Risk factors of breast cancer in Burma. *Int. J. Cancer*, **21**, 432.

TRAPIDO, E.J. (1983). Age at first birth, parity, and breast cancer risk. *Cancer*, **51**, 946.

TULINIUS, H., DAY, N.E., JOHANNESSON, G., BJARNASON, O. & CONZALES, M. (1978). Reproductive factors and risk for breast cancer in Iceland. *Int. J. Cancer*, **21**, 724.

VESSEY, M.P., MCPHERSON, K., YATES, D. & DOLL, R. (1982). Oral contraceptive use and abortion before first term pregnancy in relation to breast cancer risk. *Br. J. Cancer*, **45**, 327.

WYNDER, E.L., MACCORNACK, F.A. & STELLMAN, S.D. (1978). The epidemiology of breast cancer in 785 United States Caucasian women. *Cancer*, **41**, 2341.